# The Psychological Challenge of Late-Life Vision Impairment: Concepts, Findings, and Practical Implications

**DOI:** 10.1155/2013/278135

**Published:** 2013-04-17

**Authors:** Hans-Werner Wahl

**Affiliations:** Department of Psychological Aging Research, Institute of Psychology, Heidelberg University, Bergheimer Stra**β**e 20, 69115 Heidelberg, Germany

## Abstract

The intention is to summarize the body of evidence speaking to the psychological challenges faced by visually impaired older adults, as well as their coping efforts. This evidence is substantiated by a rich set of concepts, theories, and empirical findings that have accumulated under the umbrella of age-related psychoophthalmology (APO). I introduce the field of APO and continue with a discussion of important concepts and theories for a better understanding of adaptational processes in visually impaired older adults. I then summarize the most relevant and most recent data from four areas: (1) everyday competence, (2) cognitive functioning, (3) social functioning, and (4) subjective well-being-related outcomes, depression, and adaptational processes. Thereafter, major insights related to the current state-of-the art psychosocial interventions with visuallyimpaired older adults are reviewed. I close with the need that the public health community should become more aware of and address the psychosocial needs of visually impaired older adults.

## 1. Introduction

Loss of sight is strongly associated with chronological age and frequently used as an important marker of the awareness of getting “old” [1]. Severe vision impairment has been found in 20% of those 65 years and older and 25% of those 75 years and older [2, 3]. Age-related macular degeneration (AMD) is the leading cause of poor vision in the industrialized countries, affecting one in five people between 65 and 74 years of age and one in three people 75 years of age or older [4]. Although AMD treatment has significantly improved during recent years, currently available medical interventions can halt disease progression only in about 10 to 15% of cases [5]. Thus, the majority of older adults who are faced with AMD are psychologically challenged to adapt to ongoing and progressive visual loss, which typically occurs as deterioration of the central visual field (i.e., scotoma), affecting reading ability, overall daily functioning, and mental health [6, 7]. Furthermore, other chronic conditions, such as glaucoma, diabetic retinopathy, or diseases of the optic nerve—occurring most frequently in late life—come with the need for psychological adaptation, because treatment options may be limited. 

In this paper, it is my goal to highlight and synthesize key research speaking to the psychological consequences of age-related vision impairment and how such consequences may become a significant component of efficient treatment and rehabilitation. This includes the substantial consideration of our own research in the past 15 years. Evidence on the psychological situation of older adults with vision impairment is an important addition to ophthalmological research and treatment, and I suggest the term *age-related psychoophthalmology* (APO) to address this needed field.

## 2. Why Age-Related Psychoophthalmology Is a Needed Field

The bottom line is that demographic change and the constantly increasing life expectancy [8] have reached the center of ophthalmology and optometric day-to-day practice. Hence, the older patient is on the way to become the “standard” patient for eye care specialists. As a consequence, eye care specialists are increasingly confronted with the psychological challenges related to aging and covarying conditions, loss of sight and respective quality-of-life implications in particular, but also frequently in conjunction with other occurrences, such as loss in hearing, mobility, and cognitive function. Thus, I can hardly imagine ophthalmological treatment of the older patient only driven by an understanding of vision impairment as a circumscribed disease of the retina/fovea or another part of the optical apparatus. Instead, knowledge of the psychological situation of the older patient is also needed and may significantly help further optimize treatment and rehabilitation outcomes, as well as the efficient involvement of other disciplines, such as psychology, social work, and occupational therapy. APO adds to a long tradition of strongly psychologically framed research addressing the role and consequences of severe vision impairment in infancy [9]. In some contrast, while it is true that aging and change in normal vision function have long been a target for geropsychological research (e.g., [10]; see also [11]), psychological research on older individuals' efforts and experiences to cope with severe vision loss and needed psychosocial interventions is fairly new, having only been in existence since the end of the 1970s/beginning of the 1980s (e.g., [12] as an early study in the area).

More concretely, what are the primary goals of APO and how can this field add to the more medical part of ophthalmological treatment addressing older adults? First, APO provides findings on the full range of psychological issues involved in the experience and behavioral and emotional outcome of age-related vision impairment. Major areas include (1) everyday competence, (2) cognitive functioning, (3) social functioning, and (4) subjective well-being-related outcomes, depression, and adaptational processes. Second, APO also translates its more fundamental research insights to psychosocially framed intervention programs with visually impaired older adults with the aim to improve their quality of life in terms of behavioral and emotional functioning. Third, APO may also serve as a bridge builder between the aging-oriented areas of gerontology and geriatric medicine and ophthalmology. This includes, for example, recent concepts and theories developed in geropsychology speaking to how aging and the occurrence of chronic conditions are experienced, where psychological resilience may reach its limits, and what can be offered in a crisis situation.

At the more practical level, APO should enable eye care specialists to make better use of the existing body of evidence regarding psychosocially framed findings on age-related vision impairment in their day-to-day professional action. I see four primary reasons why such evidence emerging out of APO may make a difference in high-quality ophthalmological treatment. First, having a better understanding of everyday competence, the role of cognitive functioning, social resources, and well-being-related dynamics in visually impaired older adults may enrich the ophthalmologists' professional background knowledge of the patient. This knowledge is important for diagnostic evaluation, treatment decisions, predictions of long-term outcomes, and general recommendations aimed to inform the patient, as well as her/his family environment. For example, for an older patient with rich psychological and social resources to deal successfully with occurring vision impairment in day-to-day life, available but possibly risky treatment options—promising a limited improvement of objective vision functioning—may not be a good option. 

Second, it is also critical to have an understanding of the more fundamental mechanisms and systemic interrelations in older patients (e.g., among visual, hearing, mobility, and cognitive impairment), because such evidence helps to evaluate overall vulnerability and likely future trajectories of the patient. For example, cognitive status plays a critical role for visual functioning, and the remaining useful field of vision depends on remaining information-processing speed and not only on high functioning of the end organ [13].

Third, psychoophthalmological concepts and findings may also help to enhance the professional relationship with the visually impaired older patient; this can be a critical issue, when problematic and threatening diagnostic findings (e.g., the diagnosis of AMD) need communication and acceptance by the patient. For example, communicating the diagnosis of AMD to a very old individual already confronted with other major chronic diseases, cognitive impairment—or clear indications of loneliness—should be linked with recommendations of psychosocial support services, and such recommendations should be as concrete as possible. 

Fourth—closely related with the previous points—high-quality ophthalmological treatment may profit from detailed knowledge of available psychosocial intervention programs (to be addressed in more detail below) and their efficiency, availability, and financial implications. That is, knowing about the full scale of interventions and rehabilitation efforts evolving from psychological research with visually impaired older individuals may enhance ophthalmologists' repertoire of goal-directed consultations and recommendations for patients and caregivers, particularly in instances in which the boundary of pure ophthalmological treatment has been reached. Here, I see a huge potential of high-level multiprofessionalism that includes psychologists, occupational therapists, and social workers. Unfortunately, such highly synergetic professional collaboration is still in development but deserves much more attention in the future.

Finally, an important future task of APO is to incorporate medical-ophthalmological education and training situation for medical students and early-stage eye care specialists. Here, the major issue is to be sensitive to the various needs and challenges of “seeing the demographic revolution” in a very concrete way in one's own day-to-day practice.

## 3. Major Concepts, Models, and Theories for Age-Related Psychoophthalmology

### 3.1. Lifespan Developmental Orientation

In terms of concepts important to understand visual impairment in later life, a lifespan developmental view is a key approach. As Baltes [14] argued, lifespan developmental trajectories reveal a complex intertwining of aging, age-normative occurrences (such as retirement), and nonnormative events. Vision impairment occurring in late life reflects, in a sense, all of these elements; the likelihood of vision impairment clearly increases with age, and aging individuals expect in their late-life phase a more frequent occurrence of health instances. At the same time, severe and enduring vision loss, comes as a surprise for many older adults (e.g., “Why me?”), and there is thus also a nonnormative and critical life-event characteristic inherent in the experience of this functional-loss situation. While normative vision decrease is normally tolerated by most aging individuals as a condition affecting practically all older adults, pathological and hard-to-treat vision impairment is not; furthermore, it typically requires serious and effortful strategies of psychological adaptation. Adding to a lifespan view, research also revealed that the impact of vision loss is partially different in old age as compared to middle age. For example, change in career is not an issue for visually impaired older adults but high on the agenda of visually impaired middle-aged individuals [15].

### 3.2. Self-Regulatory Competencies

A core issue of understanding adaptational dynamics is related with the assumption that developing individuals have access to a “toolbox” of self-regulatory strategies enabling them to adjust to critical life situations. Heckhausen, and Schulz [16] argued that human kind strives from birth to death for what they have labeled *primary control*, that is, ways and means to impact one's own environment to achieve important life goals. However, aging and particularly pathological occurrences that accompany aging—such as vision impairment—challenge the fundamental motivation toward primary control and demand additional strategies to support primary-control goals. That is where *secondary control strategies,* such as disengagement from no-longer achievable goals and the reinterpretation of life priorities (e.g., “Travelling is no longer important for me.”) come into play. Secondary control should not be seen as something negative but as a means to enhance goal engagement based on remaining competencies and driven by personal needs and priorities. Therefore, the lifespan theory of control suggested by Heckhausen and colleagues is a helpful conceptual tool to understand better how visually impaired older adults cope with their situation (e.g., [17, 18]).

Brandstädter and Renner [19] have developed a similar argument in their differentiation between assimilative and accommodative coping strategies. While *assimilative strategies* indicate operating in a state of ongoing goal pursuit, the *accommodative mode* allows for flexible goal adjustment. Translated to chronic vision impairment, it seems critical for older adults with vision impairment not to psychologically give up but to keep the pursuit of goals such as autonomy and self-efficacy; at the same time, flexibly adjusting for goals no longer achievable or only achievable with high costs or failure risks seems to be highly adaptive [20–22].

### 3.3. Person-Environment Interactive Views

Vision loss brings serious challenges to one's interaction with the physical-spatial environment and, therefore, *person-environment fit models* are important in understanding the day-to-day consequences of vision impairment. Such models assume that environmental hazards, for example, bad light or floor conditions, may reveal a particularly strong negative impact when competence is low [23, 24]. Regarding the social environment, *socioemotional selectivity theory* [25] argues that older adults invest much in the maintenance of their close and intimate relationships because of a more limited future-time perspective in old age, while less intimate relationships tend to lose importance and may decrease in importance. This social environment-oriented optimization strategy may also be very helpful for visually impaired older adults. The downgrading of less significant social relationships may even be stronger, because all “social energy” is invested in the maintenance of intimate and close relationships. 

### 3.4. Maintenance of and Challenges Related to Well-Being and Cognitive Functioning

Much of what we see in terms of adaptational dynamics in visually impaired older adults is driven by the human potential for habituation and resilience. As research on the *well-being paradox of old age* (e.g., [26]) shows, all of the previouslymentioned strategies contribute to some extent to adaptation in terms of long-term restoration of well-being ([21]; see also [27], with an application to age-related vision impairment).

In parallel, however, aging is strongly affected by cognitive downward trajectories in information-processing speed, key memory functions (e.g., working memory, episodic memory), and executive control functions [28], which brings additional stress to the stability of aging trajectories under the confining condition of severe vision loss. According to Birren's [29] *cascade hypothesis*, age-related performance deficits in the sensory system may show a “domino effect” on impairment in other areas of functioning, such as cognitive abilities. The assumption is that the slackened rate of information flow to the brain due to sensory impairment produces a state of insufficient stimulation and deprivation; this, in turn, has a negative effect on cognitive performance. The *common cause hypothesis* ([30]; see also [2]) maintains that the cognitive, sensory, and motor systems increasingly dedifferentiate at the neocortex—as well as at the behavioral level—in later life. Therefore, all of these changes may echo a common cause, that is, the common aging of central brain areas, such as the frontal lobe and the visual and motor cortex. Generalizing further from such insights, it is important to acknowledge that, particularly in old-age, vision-related processes (such as speed of visual information processing, selective visual attention processes, reading capacity, or using the remaining visual field most effectively) are strongly linked with central nervous system function and are not solely rooted in the end organ, that is, the eye [10, 31].

In conclusion, adaptation to visual impairment seems to reflect in particular clarity and sharpness, what lifespan researchers have termed the gain-loss dynamics of human development [14]. On the one hand, a well-filled “tool box” of strategies is at hand to help the old individual affected by severe vision impairment to make the best out of her/his situation in terms of autonomy and well-being, because many older adults have optimized the world in which they live over the years as a highly predictable and socially stable one, they are able to counteract threats to their cognitive-emotional well-being to a large extent. However, if chronic stressors—such as severe vision impairment—can no longer be avoided or psychologically reinterpreted, high negative emotional arousal may occur, which is likely to require psychosocial intervention and wide-scale support. That said, the experience of visual impairment in old age may be seen as a prime example of reasons to learn about the interplay of strengths and vulnerabilities as people age [32].

## 4. Major Findings of Age-Related Psychoophthalmology

### 4.1. Everyday Competence

Everyday competence is a concept frequently used in geropsychology to address the day-to-day functioning of aging individuals [33]. Everyday competence includes basic and instrumental activities of daily living (ADL/IADL) but also activities covering socializing and leisure life. Age-related vision impairment has robustly been found to be closely associated with significantly lower everyday competence, because visual capacity is a critical prerequisite for such behaviors [34]. In contrast, hearing loss has been found not to have a major impact in particular on ADL/IADL [35]. Indeed, lowered everyday competence appeared as the best of a range of variables (including cognitive function and well-being-related measures) used to differentiate between visually impaired and visually unimpaired older adults [36]. Heyl et al. [37] found in a sample of community-dwelling older adults (aged 55–98 years) that vision status mediated the connection between age and out-of-home competence; therefore, the direct effect of age on everyday functions is clearly reduced after vision function is taken into consideration.

Furthermore, vision impairment affects more strongly IADL and leisure activities as compared to ADL, because the execution of IADL is more complex and depends more strongly on environmental enhancing or hindering factors, such as unexpected construction areas or the availability of low-floor bus transportation [38, 39]. Hence, shrinkage in IADL competence reflects a kind of early behavioral marker of severe vision impairment, whereas significant ADL decrease only happens later in the process of chronic vision loss.

Additional support for the critical role of IADL comes from at least two research directions. For one, Wahl et al. [40] found, when comparing severely visually impaired and visually unimpaired older adults, that those being blind and severely visually impaired (and living in apartments/houses with relatively more environmental hazards) also were lower in IADL functioning, but not in basic ADL. Wahl et al. [39] interpreted this finding as support for the assumption that visually impaired older adults invest as much as possible in the maintenance of basic ADL, even in challenging physical environments, whereas the combination of being visually impaired and living in barrier-rich environments inevitably forfeits IADL. In contrast, in visually unimpaired older adults, environmental hazards were unimportant for autonomy outcomes, which supports the core assumption of person-environment fit models [23], that is, the physical environments gain importance only when competence has fallen below a certain threshold (e.g., due to vision loss). Second, Wahl et al. [18] found that IADL (but not ADL) change in visually impaired older adults is a critical driver for change in adaptational strategies, particularly in control-strategy regulation [41]. It has been found that as long as IADL remains relatively high, visually impaired older individuals invest in maintaining goal-directed resources, whereas starting from a certain threshold of IADL competence loss, these individuals switch to secondary control strategies and disengage from no-longer-achievable goals (see also [42]).

In addition to a more general notion of ADL and IADL, it is obvious that there are two everyday competencies particularly critical for adapting to severe vision impairment. On the one hand, reading is of utmost importance for visually impaired older adults [43] and therefore a major goal in rehabilitation. Becker et al. [44] found that control-strategy use is of importance for assistive-device use such as magnifying glasses and that those being higher in primary control use assistive devices more frequently and efficiently. That is, control regulation directly or at least indirectly (via assistive devices) also contributes to the maintenance of reading. On the other hand, driving is a key competence under threat in those with severe visual impairment. The need to give up driving has generally been found to be a major psychological loss experience, intensively symbolizing decrease in autonomy and participation [45]. It is also obviously a threat to day-to-day function, particularly in countries with an underdeveloped public transport system, such as the U.S. [46]. Giving up driving has indeed been found to be a significant predictor of depression onset in older adults in the U.S. [47]. 

### 4.2. Cognitive Functioning

Linking sensory and cognitive decline as people age is an old research issue and dates back to the work of Galton [48]. At the same time, cognitive performance is also, as visual capacity, a major platform for exerting everyday competence [49]. Previous research has shown that reduced sensory function is accompanied by a decrease in cognitive performance in older adults [2]. No clear difference between the sensory modalities of vision and hearing has been identified regarding their relationship with cognitive performance, although some evidence supports that the linkage may be stronger with vision loss [50].

The work of Lindenberger and Baltes, based on the Berlin Aging Study (BASE)—but also including additional studies with a wider age range—is central in supporting a strong connection among vision, hearing, balance, and cognitive functioning in later life. The BASE [51] has been particularly noteworthy in this context, because it assessed sensory functioning in a heterogeneous sample of old and very-old participants (70–103 years of age) by means of standardized (objective) procedures covering near- and far-vision functioning, while many geropsychological studies only rely on subjective assessments. In addition, a state-of-the-art cognitive assessment battery has been applied in BASE, which means that measurement went far beyond cognitive screening measures such as the Mini-Mental State Examination, which are of limited sensitivity for detecting age-normative change in cognitive functioning. Lindenberger and Baltes [30] found with a subset of participants of BASE that general intelligence correlated just as strongly with visual as with auditory ability. In a model containing age, sensory function as well as intelligence, visual and auditory function, predicted a large portion of interindividual differences in intelligence and indeed fully mediated the negative correlation between age and intelligence. This finding has meanwhile been replicated by a number of other research groups and may be regarded as rather robust (e.g., [52, 53]). In addition, Baltes and Lindenberger [54] observed that sensory measures were better predictors of intelligence than sociostructural variables such as education or social class. They also showed that the connection among sensory functioning and intelligence was much closer in older adults as compared to adults in early and middle adulthood (see also [53]). Longitudinal data support the view that the causal dynamics involved in the vision and intelligence change connection are complex and that each of these variables can drive change in the others across longer periods of later life [55]. Henderson et al. [56] were able to show based on their long-term data that the significant correlation between vision and intelligence in a sample aged 83 years remained after controlling for child intelligence at the age of 11 years. This supports as do many of the aforementioned findings the *common cause hypothesis*. However, Henderson et al. [56] also found that childhood intelligence is able to predict visual function 72 years later, at age 83. A possible interpretation is that higher intelligence early in life leads to more investments into visual health across the life course (searching for better treatment, more preventive efforts, etc.) and therefore adds to maintaining visual status and provoking the significant correlation across an amazing time span. 

Vision status also plays a role when it comes to the connection between cognitive function and everyday competence, a linkage that is generally challenged as people age and that may lead to endpoints such as dependence from others and transition to long-term care. Heyl et al. [37] observed that the link between vision status and out-of-home everyday competence is moderated by cognitive status. In a more recent study, being able to add to the understanding of such a moderation process, Heyl and Wahl [57, 58] showed that the connection between cognitive function and everyday function is much closer in visually impaired older adults as compared to visually unimpaired older adults, which possibly means that visually impaired elders rely more intensely on their cognitive resources. Reversely, ongoing age-related cognitive decrease may bring a particularly dramatic impact in visually impaired older adults, who are already challenged in their everyday competence due to their vision loss. Causality dynamics may also work in the opposite direction. As Rovner et al. [59] observed in a study with AMD patients over 64 years of age covering a three-year observation period and two measurement occasions, that activity loss over time due to the visual loss led to cognitive decline happening between T1 and T2. This finding fits well with the more general finding in the cognitive aging literature that the exertion of social and leisure activities is important for maintaining cognitive functioning [60].

### 4.3. Social Functioning

Social relations, as well as social support, have generally been found to be of key importance for older adults [61]. We concentrate in this section on issues that may accompany the experience of age-related vision impairment and, in the next section, on linkages between social relations and adaptational outcomes. Existing evidence supports the view that social relations of older adults with severe vision impairment are not fundamentally different from sensory-unimpaired older individuals. Reinhardt [62] found that visually impaired older adults nominated, on average, 5.4 persons with intimate relations within their family network, and 3.5 persons within their friendship network, which is similar to sensory-unimpaired older adults, such as those assessed in the BASE [63]. In addition, in Wahl et al. [36] study, visually impaired older adults nominated 4.7 persons as being in the most intimate circle of their social network; the respective number in a comparison group of visually unimpaired older adults amounted to 5.2 persons, which was not statistically significantly different from the visually impaired group's mean. As has also been found, the differentiation between friendship and family support is also an important one for visually impaired older adults [62]. Additionally [64], the closest family members of visually impaired older adults are children (38%), spouses (33%), and other relatives (29%). In contrast to family relations, friendship is of voluntary nature and characterized by a strong affective base and mutuality. Research has found that because of its affective as well as instrumental support, friendship becomes increasingly important for older adults with disability [62], but friends of older adults tend to be old as well (the average age of visually impaired older adults' closest friend was 70 years in the Reinhardt and Blieszner [64] study); thus, friendship networks and their respective support are vulnerable (see also [65]). 

From the perspective of socioemotional selectivity theory [25], it can generally be expected that older adults—including sensory-impaired individuals—invest much in the maintenance of their close relationships, because of a more limited-future time perspective in old age. Wahl et al. [36] found that data among visually impaired older adults revealed even lower numbers of persons in less intimate social domains as compared to visually unimpaired older adults; this suggests that socioemotional selectivity toward more intimate social partners may even be more pronounced in visually impaired older adults. Furthermore, although visual impairment may further increase the likelihood of experiencing loneliness [66], the majority of studies found visually impaired older adults not to be dramatically more affected by the experience of loneliness than visually unimpaired older adults [36, 67]. Visually impaired older adults are also particularly challenged to reestablish their social networks following vision loss, because social network members may have a tendency to sever social ties with visually impaired older adults, for example, because previous joint activities have become more difficult. As Wang and Boerner [68] observed, two strategies seem prevalent in visually impaired older adults: namely, to readjust one's behavior to maintain relationships or to let relationships go to some extent. The latter fits in a sense with Wahl et al.'s [36] finding of lower number of persons in the periphery of social networks of visually impaired older adults as compared to visually unimpaired individuals.

Particularly critical features of social partners are dependence-inducing behavior, the lack of an independence-supporting attitude, and overprotection [69]. Perceived overprotection in visually impaired older adults is on the rise, wherein functional disability and received instrumental support increases [70]. Also, perceived overprotection increases over time and has been found to be unaffected by rehabilitation service use [71]. In general, it seems a challenge to provide visually impaired older adults with the needed instrumental support while at the same time avoiding to underestimating remaining capabilities [72]. Overprotection may put constraints on the visually impaired older adults' “true” functional capacity and thereby contribute to loss in competence in the longer run due to disuse [69, 73]. 

### 4.4. Subjective Well-Being-Related Outcomes, Depression, and Adaptational Processes

Subjective well-being (SWB) is frequently defined via its cognitive component as degree of satisfaction with one's current life. Many studies with visually impaired older adults have found that with age-related vision loss comes a significant decrease regarding the cognitive component of well-being [39, 67]. As has also been observed, the discrepancy in SWB between visually impaired and visually unimpaired older adults tends to persist over time [38, 74]. However, Pinquart and Pfeiffer [75] found in their meta-analysis of 198 studies that the effect size regarding differences in vision-unspecific measures (such as general well-being or positive/negative affect scales) was small, whereas larger negative effects regarding well-being appeared in vision-specific measures of quality of life (such as the NEI VFQ-25; [76]). In general, widening the picture of adaptation to visual impairment with the consideration of everyday competence, robust evidence underscores that differences in well-being-related indicators—including constructs such as self-acceptance, positive relations with others, and tone of future-time perspective between visually impaired and visually unimpaired older adults—are clearly less pronounced than differences in impairment in ADL-IADL status and leisure activity level [36, 77]. This suggests that although vision impairment is a pronounced psychological challenge in late life, many visually impaired older adults seem to adapt rather well at the level of cognitive well-being, thus also supporting what has been titled the paradox of well-being in later life [26].

At the same time, it is critical to acknowledge that visually impaired older adults represent an at-risk population, in which the positive impact of human adaptation and the drawback of reaching the limits of psychological resilience go hand in hand. Affect balance (ratio of positive and negative effect) has been found to be more toward the negative pole in visually impaired older adults [77] and depression has consistently been found to be significantly increased in visually impaired older adults [78–80]. Rates identified in epidemiological studies roughly vary between 15% and 30% and are particularly high in AMD patients [78]. This is also important, because depressive symptoms may accelerate both cognitive decline and decline in everyday competence in AMD patients [81].

In addition to depressive mood, maintenance and decline in positive effect are important for adaptation. Schilling and Wahl [27] found that positive effect goes down in the period after having received the diagnosis of AMD, but then also increases again after 2–4 years. Thereafter, rather constant decrease has been observed, probably due to the progressive course of AMD in combination with growing comorbidity and other loss occurrences (such as widowhood) as people age further. Control regulation, as argued by Heckhausen et al. [41], also seems important for positive effect. Wahl et al. [17] observed in a respective analysis that compensatory control strategies (called “compensatory primary control” by Heckhausen et al., [41]), such as involving the help of others as well as strengthening one's commitment to important life goals (called “selective secondary control” by Heckhausen et al., [41]) coincides with positive effect, whereas the effect of exerting the impact on one's environment (called “selective primary control” by Heckhausen et al., [41]) on positive effect was fully mediated by ADL-IADL and a vision-specific adaptation measure (called adaptation to vision loss; [82]). The critical role of maintaining everyday competence (and not objective vision impairment) for well-being and adaptation to vision loss in visually impaired older adults has also been confirmed in other research [83], including the transition from assimilative to accommodative coping [19, 20]. Decrease in IADL competence also seems to drive the transition to modes of control regulation connected with goal disengagement and flexible goal adjustment, which Heckhausen et al. [41] have labeled “secondary compensatory control” ([18]; see also [84]). 

In addition to change in self-regulation as a significant adaptational process, social resources have been found to play a critical role in adaptation to vision loss and other well-being-related outcomes—cross-sectionally as well as longitudinally—and family and friends provide distinct contributions to the maintenance of well-being [62, 85]. Perceived overprotection may also lead to negative consequences in terms of heightened depression and anxiety over time [73]. 

### 4.5. A Glance on Dual Sensory Impairment

Dual sensory impairment affects one-fifth of those 70 years of age and older [86] and therefore also deserves attention. Previous research supports the notion that the overall psychosocial situation of those with dual sensory impairment is even worse as compared to those with sole vision impairment, particularly in the area of everyday functioning [86]. In addition, higher rates of depression and lowered well-being have also been found in those affected by dual sensory loss [87]. 

## 5. Psychosocially Framed Intervention Research with Visually Impaired Older Adults: Promises and Unknowns

The research summary provided in the previous section underscores that the experience of age-related visual impairment comes with pronounced loss, although the capacity to maintain subjective well-being and positive effect has to be acknowledged at least in a considerable portion of visually impaired older adults. Overall, I come to the conclusion that such naturally occurring psychological resilience and reserve capacity to counteract the threat elicited by the experience of chronic vision impairment are surprisingly high; on the other hand, given the vulnerability of visually impaired older adults—particularly, the “alarmingly high” ([78, p. 591]) rate of depression—qualified professional support of visually impaired older adults in the psychosocial domain with the potential to significantly add to the best available ophthalmological treatment seems to be a *must*. 

Wahl et al. [88] analyzed 15 intervention studies, with a considerable subportion of randomized, controlled intervention trials. Most interventions could be characterized as self-management and disease management-like efforts and are promising for visually impaired older adults. Major elements of such programs include stress-reducing strategies (e.g., muscle-relaxation exercises), goal-directed problem-solving strategies to evoke positive affect, activating available resources, and information and consultation. Typically, such programs are conducted in a group format in an eye clinic, bringing together 6–8 visually impaired older adults, for weekly sessions of 2-3 hours, over 6–8 weeks. Group sessions are moderated by a clinical psychologist with particularly professional experience with older adults. 

A case example emerging from our work in this area [89, 90] may illustrate what can be achieved. Mrs. A., 79 years of age, who had always been “single”—without many family ties—had been a traveling person throughout her life. After retirement from her job as an assistant to the director of a university library, her plan was to intensify her “world traveling.” However, a rapidly developing AMD occurrence precluded this ambition. Mrs. A. became, to her own surprise, very depressed and, to a large extent, unable to manage her daily affairs. In the group sessions of the self-management program, of which she learned in the practice of her eye doctor, she remained rather quiet and obviously in a depressed mood throughout the first three sessions. In the fourth week's session, she reported having learned muscle relaxation and also felt somewhat better. The session was then also used to plan visiting a restaurant—an activity she had not done in a year—although she always found having lunch in a restaurant very enjoyable. In particular, all the steps to successfully conduct this activity were discussed and preplanned. Later in the flow of the program, Mrs. A. reported of two successful lunch events and said that she had been very proud of herself. She had also begun to reinstate phone conversations with some of her friends and indeed started to plan a 7-day holiday together with her best friend. Although she still reported being frequently in a depressed mood at the end of the 8-session program, she also felt an increasing feelings of mastery in her day-to-day life. She also had begun to disengage from her “world travel” goals and increasingly thought about remaining possibilities much enjoyable for her.

Positive effects have been reported in such self-management programs regarding depression, increased well-being, self-efficacy, and stress reduction. Dose of intervention is important (too-short and less-intensive programs may indeed elicit negative effects; [90]). More recent work provides additional support for the assumption that self-management programs are useful [91, 92] and add to the need to seriously consider such programs as a standard health treatment. Still, many of the available studies operate on small samples and include a number of other methodological limitations, which means that larger high-quality studies are an important need in the area [93]. It is also obvious that such programs should find a strong liaison with classic high-caliber rehabilitation programs, including effective reading training [43]. Given the findings on the role of cognitive resources in visually impaired older adults (see respective section above), cognitive training may be an important addition to psychosocial intervention and rehabilitation [58, 94]. Furthermore, physical training programs, which have proven efficient with old and very old individuals—including those being cognitively vulnerable—may also be of significant advantage for visually impaired older adults. As has been found, such programs not only increase posture, gait, and general physical fitness, but also they prevent falls and enhance well-being, self-efficacy, and cognitive function, especially executive control [95]. Finally, it may be important to consider personality traits of visually impaired older adults so as to achieve the best psychosocial outcomes possible [96]. In summation, I would recommend multi-component interventions as the best available psychosocially framed interventions for visually impaired older adults. Such multicomponent interventions that not only offer direct psychosocial guidance (as described previously), but are also closely intertwined with cognitive and physical engagement and independence training in ADL.

Lastly, emerging evidence supports the notion that psychosocially framed interventions may also contribute to saving health costs (e.g., via reduced psychopharmacy) and may also enhance commitment toward secondary prevention, for example, avoiding later treatments of depression [97]. 

## 6. Outlook

Severe vision loss and blindness have always been an issue going far beyond the mere functional loss and respective medical-ophthalmological treatment into culture, society, and fundamental questions concerning the human condition. The prophet *Tiresias* in Greek mythology was blind but also was able to offer wise recommendations and helpful forecasts. The idea that blindness may bring man to fundamental insights, new “ways of seeing”, and extraordinary achievements has persisted through cultural history. The secular trend version of this cultural history comes with the more recent significant expansion of life expectancy, increasingly making the older adult the standard patient of eye care specialists. APO now provides a rich portfolio of research supporting the view that many older adults continue, in a sense, in the Tiresias tradition and are able to make the best out of their vision impairment. However, major subgroups of older adults with vision impairment also are in need of high-quality professional support to improve their psychosocial adaptation. Although promising intervention research has also been generated within APO, such psychosocial support services currently seem to be more the exception than the usual or even standard professional offer in the USA, as well as in Europe or other regions of the world. This is disappointing and requires that the multiprofessional treatment and rehabilitation of visually impaired older adults should find high priority in public health terms in the future.
